# Biological applications of graphene-based nanomaterials in neurodegenerative diseases

**DOI:** 10.1016/j.mtbio.2025.102064

**Published:** 2025-07-09

**Authors:** Rong-kun Zhu, Jun Shi, Hong-jian Zhou, Ling Ge, Wen-hao Yin, Hui Zeng, Xiong-wei Wang

**Affiliations:** a Department of Neurosurgery, Yangpu Hospital, School of Medicine, Tongji University, Shanghai, 200090, China; b Department of Neurology, Yangpu Hospital of Traditional Chinese Medicine, Shanghai, 200090, China

**Keywords:** Graphene-based nanomaterials, Neurodegenerative diseases, Alzheimer's disease, Parkinson's disease, Amyotrophic lateral sclerosis

## Abstract

Neurodegenerative diseases (NDDs) have become a major challenge in global public health due to neurotoxicity caused by progressive neuronal degeneration and abnormal protein aggregation, which has attracted widespread attention. Graphene-based nanomaterials provide innovative solutions for the early diagnosis and precise treatment of NDDs by virtue of their ultra-high conductivity, large specific surface area and multifunctional properties. In this paper, we systematically discuss the key applications of these materials in the diagnosis and treatment of NDDs, and deeply analyze the technological breakthroughs and clinical translation challenges. The core of this paper is to illustrate that graphene-based nanomaterials are expected to reshape the paradigm of NDDs diagnosis and treatment through cross-scale technological innovations, promoting the synergistic development of early diagnosis, personalized treatment and real-time monitoring, and providing a transformative strategy for overcoming NDDs.

## Graphical abstract

**Figure:**
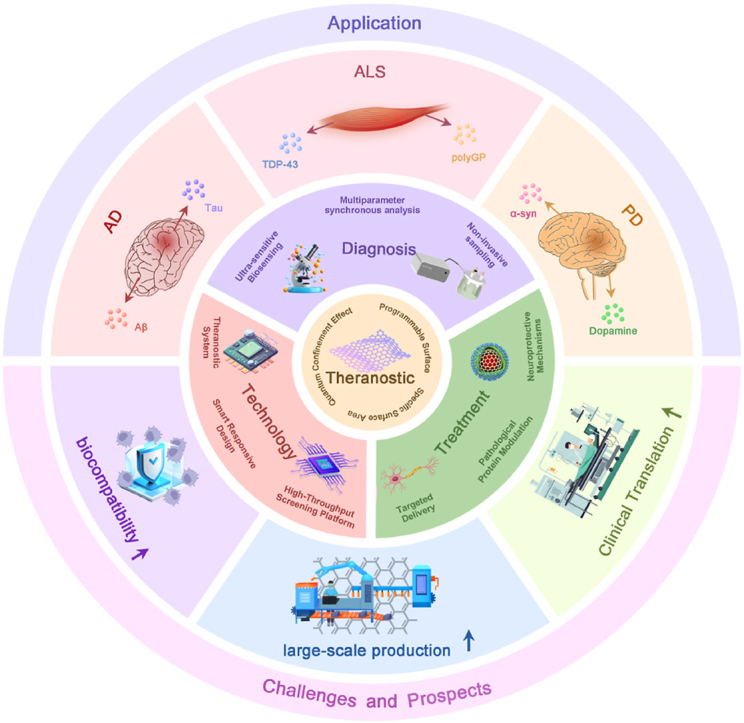

## 1 Introduction

Neurodegenerative diseases (NDDs) are multifactorial pathological processes characterized by progressive functional deterioration of the central nervous system, with core pathological manifestations involving protein homeostasis dysregulation leading to abnormal aggregation and fibrillary damage [1]. Studies demonstrate that key molecules including β-amyloid (Aβ), tau protein, and α-synuclein (α-syn) undergo aberrant post-translational modifications or degradation pathway impairments, forming oligomeric intermediates that ultimately transform into β-sheet-rich amyloid fibrillar deposits [[2], [3], [4]]. These pathological alterations trigger cascading cellular events such as mitochondrial energy metabolism dysfunction, endoplasmic reticulum stress, and reactive oxygen species (ROS) overproduction, culminating in synaptic plasticity loss, neural network disruption, and neuronal apoptosis [5,6]. Based on distinct anatomical involvement and neuropathological signatures, the NDDs spectrum encompasses Alzheimer's disease (AD), Parkinson's disease (PD), Huntington's disease (HD), Amyotrophic lateral sclerosis (ALS), and frontotemporal lobar degeneration [7]. Each subtype exhibits characteristic biomarkers: AD presents with Aβ plaques and hyperphosphorylated tau neurofibrillary tangles [8,9], PD demonstrates α-syn-containing Lewy bodies [10], while ALS correlates with TDP-43 proteinopathy [11]. Epidemiological data reveal AD and PD as the two most prevalent NDDs, affecting approximately 55 million and 6 million individuals globally, respectively [12,13]. The substantial health burden and socioeconomic impact of these disorders pose critical public health challenges, necessitating novel diagnostic and therapeutic strategies.

Recent advancements in two-dimensional nanomaterials have opened new avenues for NDDs management, with graphene-based nanomaterials (GBNs) emerging as a research focus due to their unique physicochemical properties [14,15]. These materials exhibit exceptional electrical conductivity, superior biointerface compatibility, and blood-brain barrier penetrability [16]. Their surface charge distribution can mimic neuronal electrophysiological microenvironments to promote neurite outgrowth and axonal guidance [17] functionalization strategies, GBNs can be engineered as composite scaffolds carrying neurotrophic factors or therapeutic agents, synergistically providing mechanical support and biochemical regulation to enhance neural progenitor cell differentiation [[18], [19], [20]] evidence indicates that surface-modified graphene quantum dots (GQDs) significantly improve drug transmembrane transport efficiency through nanoscale size effects and tunable surface chemistry [16,21]. In prevalent NDDs including AD, PD, and ALS, GBNs demonstrate therapeutic advantages through their distinctive electronic, optical, thermal, and mechanical properties to improve clinical outcomes [[22], [23], [24], [25], [26]]. Clinically, GBNs present multidimensional applications: graphene-based biosensors utilizing field-effect transistor principles achieve picomolar-level detection of phosphorylated tau and Aβ 42 in AD cerebrospinal fluid [[27], [28], [29]]; Therapeutically, functionalized graphene oxide (GO) impedes AD progression by inhibiting Aβ fibrillization [16], while GQDs exert neuroprotection in PD through radical scavenging and α-syn aggregation suppression [30]. These breakthroughs highlight GBNs' potential in precision diagnosis and treatment of NDDs, establishing their investigation as a pivotal research direction in contemporary neurology. This review systematically summarizes recent advances in GBNs applications for early diagnosis and therapeutic intervention of major NDDs.

## 2 Graphene-based nanomaterials

### 2.1 Graphene-based nanomaterials

As a novel two-dimensional carbon-based functional material, graphene features a two-dimensional honeycomb arrangement of carbon atoms through sp^2^orbital hybridization, forming a single-atom-layer hexagonal lattice system with exceptional mechanical properties and nanoscale thickness [31,32]. Notably, while pristine graphene maintains an intact crystal structure, its marked surface hydrophobicity substantially compromises dispersion stability in aqueous environments, posing a critical challenge for biomedical applications. To address this limitation, researchers have developed chemically modified derivatives such as GO and its reduced form reduced graphene oxide (rGO), which preserve fundamental characteristics while significantly enhancing colloidal stability and surface functional group density - essential prerequisites for biomedical utilization [33,34]. Based on carbon layer stacking configurations, these materials can be systematically classified into four structural categories: single-layer graphene (SLG), few-layer graphene (FLG, 2–5 layers), multi-layer graphene (MLG, 5–10 layers), and graphite-like nanoflakes (>10 layers) [35,36].

In terms of physicochemical properties, this material family exhibits multifaceted advantages: its extraordinary electron mobility provides a fundamental platform for developing high-precision sensing components, while the theoretical specific surface area of 2630 m^2^/g positions it as an exceptional candidate for molecular adsorption and interfacial catalysis [37]. These inherent characteristics have driven multidimensional innovations in biomedical applications, encompassing gene delivery vector design, antibacterial interface construction, smart drug delivery systems, ultrasensitive biodetection, molecular imaging enhancement, and tissue engineering scaffold fabrication [[38], [39], [40]]. Strategic surface engineering through functionalization approaches significantly improves biological compatibility and targeted delivery precision, which proves crucial for developing integrated theranostic platforms. Of particular interest are zero-dimensional GQDs, whose tunable fluorescence emission, quantum size effects, and low cytotoxicity render them uniquely valuable in precision medicine applications such as disease biomarker detection and in vivo tracking [41]. With breakthroughs in advanced fabrication techniques and deepened understanding of structure-activity relationships, graphene-based nanosystems are poised to overcome limitations of conventional diagnostic and therapeutic technologies, evolving into next-generation medical tools with intelligent responsiveness and molecular recognition capabilities - thereby injecting new momentum into modern medical technology innovation. The following table is a comprehensive comparison of graphene-based nanomaterials (Table 1).

**Table 1:** Comprehensive comparison of graphene-based nanomaterials: Chemical derivatives vs. structural configurations.

Material	Characteristics	Advantages	Limitations	Applications	Primary Mechanisms	Refs
**GO**	Abundant O-groups (-OH/-COOH); hydrophilic; layered	High BET; facile functionalization; good biocompatibility	Low conductivity; structural defects; debated toxicity	Drug delivery; biosensing; antimicrobials	Covalent conjugation; π-π stacking; biomolecule adsorption	[31,32]
**rGO**	Reduced O-groups; restored conductivity; hydrophobic	High conductivity; strong NIR absorption; good mechanical strength	Aggregation tendency; potential cytotoxicity	Electrochemical sensors; photothermal therapy; electrodes	Enhanced electron transfer; photothermal conversion; electrode modification	[33,34]
**GQDs**	<10 nm; tunable fluorescence; quantum confinement	Low cytotoxicity; intrinsic fluorescence; cellular uptake; good biocompatibility	Low quantum yield, surface chemical complexity; synthesis complexity	Bioimaging; sensing; drug delivery	Fluorescence tracking; enhanced tissue penetration; surface-mediated sensing	[39]
**GNRs**	Quasi-1D ribbon; tunable width/edge; size-dependent bandgap	Tunable semiconductivity; unique edge effects; high mechanical strength	Complex synthesis; high cost; scalability issues	High-sensitivity sensing; nanoelectronics	Edge-dependent electron transport; High aspect ratio enhances cellular interaction	[42]
**Functionalized** **GO**	Polymer/peptide/antibody-modified surfaces	Enhanced stability; prolonged circulation; active targeting	Masking of intrinsic properties; complex preparation	Targeted delivery; theranostics	Ligand-receptor targeting; integrated therapy/diagnosis	[38,43]
**SLG**	Strictly monolayer; no interlayer interactions	Highest charge mobility; complete 2D confinement	Mechanically fragile; difficult to produce defect-free	Fundamental studies; high-precision sensors	Pure 2D electron transport; quantum effects	[44]
**FLG(2–5 layers)**	Weak interlayer coupling; layer-dependent properties	Balanced properties; maintains 2D characteristics; easier production	Property variations with exact layer number	Flexible electronics; sensors	Tunable bandgap; layer-modulated conductivity	[35]
**MLG(5–10 layers)**	Transitional properties; approaching bulk behavior	Higher mechanical strength; more stable	Losing distinctive 2D properties	Structural composites; coatings	Interlayer charge transfer; stacked π-system effects	[45]
**Graphitic Nanosheets (>10 layers)**	Quasi-bulk behavior; strong interlayer bonding	Easiest to produce; highest mechanical robustness	Essentially 3D material; no quantum confinement	Batteries; reinforced materials	Bulk-like conduction; intercalation chemistry	[46]

### 2.2 GBNs-mediated drug delivery

Graphene-based nanostructured drug delivery platforms demonstrate revolutionary advantages in smart drug delivery system development, leveraging their unique two-dimensional atomic arrangement, programmable interfacial properties, and extraordinary specific surface area [47,48]. Compared with conventional delivery systems, these platforms exhibit exponential improvements in mass-specific drug loading efficiency. Their surface-abundant reactive groups enable controlled therapeutic molecule loading and programmed release through directional conjugation techniques [49]. Crucially, synergistic regulation of covalent and non-covalent interactions within these systems significantly enhances structural stability and tissue selectivity of drug-loaded complexes. Representative strategies include π-π stacking interactions for planar drug molecule immobilization and charge complementarity-driven loading of polar bioactive compounds [50].

In overcoming biological barriers, these nanosystems demonstrate exceptional transmembrane transport efficiency. For central nervous system therapeutics, the stringent filtration mechanism of the blood-brain barrier (BBB) substantially impedes effective neuroprotective drug delivery [30,51,52]. Experimental evidence confirms that surface-engineered graphene derivatives - through topological optimization via polyethylene glycol or targeting peptide conjugation - effectively modulate hydrophilicity-lipophilicity balance and biointerface compatibility. This optimization prolongs circulation half-life and enhances BBB penetration efficiency. A landmark study demonstrated that size-controlled GQDs with tunable surface charge increased Alzheimer's therapeutic drug accumulation in lesion sites by 3.6-fold [53]. Molecular dynamics simulations further reveal specific molecular recognition between functionalized graphene nanosheets and phospholipid bilayers, with these dynamic interactions significantly promoting transmembrane drug transport while maintaining BBB integrity [54].

Regarding release kinetics modulation, the material's multimodal responsiveness enables novel precision medicine approaches. Unlike traditional carriers prone to burst release phenomena, graphene-based systems achieve pathology-driven intelligent drug release through environmental response units such as pH-sensitive linkages or enzymolytic active sites. For instance, GO-drug complexes demonstrate selective epoxy group cleavage in tumor microenvironments, enabling on-demand chemotherapeutic agent release. This spatiotemporally controlled release pattern enhances antitumor efficacy by 2.3-fold compared with conventional liposomal systems [49].

## 3 Alzheimer's disease (AD)

AD, recognized as a global public health crisis, imposes an exponentially growing socioeconomic burden. The disease's characteristic neuropathological hallmarks manifest as dual pathological deposits: extracellular Aβ aggregates forming senile plaque cores in the neuronal interstitial space, and intraneuronal neurofibrillary tangles composed of hyperphosphorylated tau protein that induce cytoskeletal disintegration [55]. Epidemiological projections indicate the AD patient population is expanding at an annual rate of 3.1 %, with global prevalence predicted to exceed 130 million by 2050 [12]. Confronting the escalating challenge of population aging, achieving early detection and disease progression monitoring has become imperative. In this context, innovative graphene-based biosensing platforms have emerged as a critical direction for transcending conventional diagnostic limitations [56].

Graphene's distinctive interfacial properties – including exceptional electrical conductivity, biointerface affinity, and ultrahigh detection sensitivity – provide revolutionary solutions for ultra-early detection of AD biomarkers [57]. Advanced studies demonstrate that three-dimensional micro-nanoarchitectured graphene sensing interfaces enable specific capture of Aβ oligomers and phosphorylated tau proteins in cerebrospinal fluid, achieving detection sensitivity improvements of 2–3 orders of magnitude compared with traditional ELISA techniques [[58], [59], [60]]. This technological breakthrough not only substantially reduces clinical detection thresholds but also establishes reliable technical foundations for developing preclinical AD diagnostic criteria.

### 3.1 The importance of graphene-based biosensors in the early diagnosis of Alzheimer's disease

Graphene's innovative applications in biosensing have revolutionized early AD detection through breakthrough technological pathways. The material's exceptional surface properties and superior conductivity establish it as an ideal substrate for constructing high-precision detection platforms. Researchers have developed graphene oxide composite electrodes for dynamic monitoring of Aβ oligomers, demonstrating two orders of magnitude sensitivity enhancement compared with conventional immunoassays [61]. Notably, synergistic modification strategies incorporating noble metal nanostructures with conductive polymers significantly amplify electron transfer efficiency at sensing interfaces. Experimental validation shows 78 % enhancement in electrochemical response signals toward Aβ monomers using these hybrid electrodes, laying the foundation for ultrasensitive diagnostic devices [62].

A groundbreaking advancement lies in the development of multiplex detection capabilities using three-dimensional graphene sensing architectures. Unlike conventional single-analyte systems, state-of-the-art vertically printed electrochemical sensor arrays enable simultaneous quantification of four critical biomarkers (Aβ40, Aβ42, total tau, and p-tau181) in blood samples, achieving 400 % increase in throughput while maintaining picomolar detection limits [63]. This integrated detection paradigm provides technical support for establishing multidimensional AD diagnostic models. From a material performance perspective, graphene's quantum confinement effects and surface electronic state modulations directly govern sensing capabilities. Research teams employing solution-gated field-effect transistor architectures have accomplished femtogram-level detection of p-tau217 protein through graphene work function engineering, demonstrating linear response across six orders of magnitude [59]. Crucially, magnetoresponsive nitrogen-doped graphene systems reduce Aβ42 detection costs by 62 % while achieving broad dynamic detection ranges, demonstrating significant advantages for clinical-scale applications [64].

Technological innovations include differential pulse voltammetry platforms utilizing atomically smooth graphene interfaces, achieving unprecedented Aβ(1–42) detection limits with strong correlation to in vivo imaging data [65]. Clinical validation studies reveal 98.7 % accuracy in serum Aβ42 detection, representing 3.2-fold sensitivity improvement over current standard methods – a milestone in non-invasive diagnostic technology advancement [60]. Next, the application of two core pathological markers (Aβ and tau proteins) to detect Alzheimer's disease is described in the following two paragraphs.

#### 3.1.1 Contribution of GBNs to beta-amyloid detection in Alzheimer's disease patients

Pathological Aβ deposition has been conclusively established as the central pathogenic driver of neuronal degeneration in AD etiology. Oligomeric Aβ species disrupt synaptic plasticity and trigger oxidative stress cascades, culminating in neuronal apoptosis and neural network destabilization [12] pathophysiological features underscore the imperative for developing high-fidelity dynamic monitoring systems, where real-time tracking of Aβ flux dynamics not only deciphers disease mechanisms temporally but also creates critical windows for pre-symptomatic intervention.

Graphene's two-dimensional nanostructure, characterized by unparalleled electrical conductivity, ultrahigh surface-to-volume ratio, and biocompatibility, positions it as a paradigm-shifting substrate for advanced biosensing interfaces [66]. Compared to conventional diagnostic paradigms dependent on mass spectrometry or ELISAs, graphene-based sensing platforms demonstrate transformative capabilities. A prototypical electrochemical aptasensor employing GQDs achieves Aβ quantification across an unprecedented dynamic range, exhibiting 100-fold enhanced sensitivity over conventional methodologies [67]. Particularly noteworthy is the development of thermally reduced rGO matrices that achieve sub-femtomolar detection limits, enabling clinically actionable ultra-early diagnostic thresholds [68].

Mechanistic investigations reveal GO exhibits dual modulatory roles in Aβ metabolism: augmenting microglial phagocytic clearance via autophagy-lysosomal pathway potentiation, and preserving neuronal integrity through mTOR signaling axis downregulation [69]. This bidirectional material-biology crosstalk unveils novel therapeutic avenues. Clinical validation studies demonstrate strong concordance between graphene-enhanced electrochemical immunosensors' salivary Aβ measurements and cerebrospinal fluid reference standards, constituting a watershed advancement in non-invasive diagnostics [70]. Despite the significant progress made, several technical bottlenecks still need to be overcome: non-specific adsorption of biomolecules affects detection selectivity, and the lack of material stability in complex body fluid environments restricts the ability of long-term monitoring. Recent studies have shown that the anti-interference performance and long-term cycling stability of sensors can be effectively improved by constructing a biomimetic phospholipid bilayer-modified interface or introducing molecular imprinting technology. These technological breakthroughs will accelerate the transformation of graphene sensing systems from laboratory to clinical applications.

#### 3.1.2 GBNs in tau protein monitoring in Alzheimer's disease patients

In the pathological progression of AD, aberrant phosphorylation of tau protein serves not only as a central driver of neuronal degeneration but also as a molecular switch governing synaptic dysfunction and programmed cell death. This microtubule-associated protein primarily localizes to axonal microtubules, where it physiologically stabilizes microtubule networks and regulates axonal transport. Pathological hyperphosphorylation induces tau detachment from microtubules and subsequent neurofibrillary tangle formation, a hallmark AD pathology [71]. The development of ultrasensitive detection platforms to monitor tau conformational changes holds significant clinical value for early AD warning and disease progression tracking.

Graphene's interfacial electronic properties and molecular recognition capabilities provide a physical foundation for advancing tau detection technologies. Recent breakthroughs involve multimodal sensing interfaces: graphene-based sensors employing entropy-driven strand displacement reactions integrated with DNA recognition elements and signal amplification systems achieve femtomolar-level tau detection in cerebrospinal fluid. Notably, synergistic effects between noble metal nanostructures and graphene enhance interfacial electron transfer efficiency, improving serum tau detection signal-to-noise ratio by 5.8-fold while reducing nonspecific adsorption by 92 % [72].

Multiplex detection systems using three-dimensional graphene sensing arrays enable simultaneous quantification of AD biomarkers. Experimental data confirm these platforms can simultaneously quantify tau phosphorylation sites (p-tau181, p-tau217), Aβ42 oligomers, and glutamate concentration fluctuations, achieving 300 % higher throughput than single-analyte systems - a critical advancement for establishing multidimensional AD diagnostic models [73]. Furthermore, impedance biosensors incorporating rGO-gold nanoparticle composites demonstrate excellent reproducibility and recovery rates in clinical validation, with high consistency between cerebrospinal fluid and serum samples 。These technological advances highlight three key advantages of graphene-based sensing systems in AD precision medicine: (1) ultrasensitivity enabling preclinical detection, (2) multiparametric analysis reflecting dynamic disease progression, and (3) non-invasive sampling enhancing clinical applicability. Integration of surface functionalization strategies with microfluidic technologies positions these systems as core tools for comprehensive AD management.

### 3.2 GBNs in Alzheimer's disease neuroprotection and drug delivery

Breakthrough advancements in graphene-based nanosystems are revolutionizing brain-targeted drug delivery and neuroprotective strategies for comprehensive AD treatment. The material's interfacial properties – including a theoretical specific surface area of 2630 m^2^/g and electron mobility exceeding 15,000 cm^2^/V·s – establish it as an ideal matrix for constructing intelligent delivery vectors [74]. Its drug-loading superiority manifests through three-dimensional advantages: (1) nanoscale architecture enables ultrahigh active surfaces for dense drug molecule loading; (2) programmable surface chemistry stabilizes drug complexes via π-π stacking and hydrophobic interactions; (3) amphiphilic characteristics enhance BBB penetration through receptor-mediated transport, achieving 3-5-fold improvement in cerebral drug bioavailability [75].

In neuroprotection, GBNs demonstrate unique molecular regulatory capacities: quenching free radicals to alleviate oxidative damage, modulating Bcl-2/Bax expression ratios to suppress mitochondrial apoptosis, and activating the Nrf2/ARE pathway to enhance cellular antioxidant defenses [76]. Notably, multifunctional nanocomposite systems exhibit synergistic therapeutic effects – gold nanoparticle-decorated GO drug carriers achieve 2.8-fold Aβ clearance enhancement while increasing neurotrophic factor secretion by 160 % in AD cellular models [74].

Despite GBNs' promising AD applications, critical challenges require resolution to facilitate clinical translation. First, sensor stability and reproducibility must be enhanced, as current systems experience signal attenuation and interference in complex biofluids, necessitating surface charge engineering and functional group optimization [77]. Second, cost-effective manufacturing processes need development to address current limitations of complex fabrication protocols and expensive raw materials. Future research should prioritize: multiplex biomarker detection systems for comprehensive early diagnosis, and innovative drug loading/release mechanisms to enable systemic AD therapeutic strategies. Here's a picture of GBNs for biological applications in AD (Table 2).

**Table 2:** Graphene-based nanomaterials for biological applications in Alzheimer's disease.

Material	Therapeutic Mechanisms	Diagnostic Applications	References
GO	Inhibition of Astrocytes and Microglia; Foster neurogenesis; Inhibit Aβ aggregation; Protect neurons; Decrease Aβ fibrillization	Detection of Aβ; Detection of Tau; Enhanced AD detection sensitivity; Biosensor for AD detection	[16]
WS_2_NS	Mitigate neuronal damage; Protect neurons; Inhibit Aβ aggregation;	Detection of Aβ; Detection of miR-29a	[78]
GO + Au NPs	Protect synapses; Inhibit Aβ aggregation;	Enhanced detection sensitivity; Decrease the detection limit	[58,63]

## 4 Parkinson's disease (PD)

PD, a prevalent neurodegenerative disorder within the NDDs spectrum, is characterized by progressive loss of dopaminergic neurons in the basal ganglia, with a protracted prodromal phase (10–15 years) preceding motor symptom onset that poses substantial challenges in clinical differential diagnosis [79,80]. Against the backdrop of global population aging, PD prevalence is escalating at an annual rate of 4.7 %, driving technological innovation toward prodromal detection paradigms. Breakthroughs in nanomedicine and biosensing technologies offer transformative approaches for PD precision medicine, where two-dimensional graphene nanomaterials emerge as game-changers in biomarker detection through their exceptional electron mobility and surface plasmon resonance effects [81]. Crucially, graphene-based sensing interfaces leverage quantum confinement effects and molecular recognition capabilities to overcome limitations of conventional detection methods. Three-dimensional graphene microelectrode arrays have achieved femtomolar-level detection of dopamine metabolites in cerebrospinal fluid, demonstrating three orders of magnitude sensitivity enhancement over traditional voltammetry techniques [82]. These advancements not only enable biomarker identification during presymptomatic PD stages but also establish the technological foundation for a novel "molecular staging-precision intervention" diagnostic framework.

### 4.1 Role of GBNs in the early diagnosis of Parkinson's disease

PD, a major neurodegenerative disorder, is pathologically characterized by selective degeneration of dopaminergic neurons in the substantia nigra pars compacta andα-syn aggregation forming Lewy bodies [83]. This degenerative process extends beyond the dopaminergic system to involve cholinergic and serotonergic neurotransmitter networks, creating complex pathological cascades that challenge therapeutic strategy development. Recent breakthroughs reveal that detecting α-syn conformational changes in cerebrospinal fluid exosomes or gut microbiome-derived metabolites (e.g., abnormal short-chain fatty acid profiles) enables PD prediction 5–7 years prior to motor symptom onset [84].

In biosensing innovation, laser-induced graphene (LIG) microelectrode arrays achieve breakthrough applications in gut-brain axis monitoring. This technology establishes prodromal PD biomarker correlation models by synchronously detecting colonic α-syn oligomers and serum dopamine metabolite fluctuations [85]. Notably, gold nanoparticle-functionalized GO carriers enhance α-syn inhibitor delivery efficiency to 75 % while providing neuroprotection via microglial TLR4/NF-κB pathway inhibition [86]. Electrochemical sensing advancements demonstrate remarkable performance enhancements: nitrogen-doped graphene/carbon nanotube heterojunctions achieve 0.1 nM dopamine detection sensitivity with 8.2-fold selectivity improvement over conventional electrodes [87]. SilvaNeto et al. demonstrated enhanced sensor conductivity and biomarker binding capacity through functionalized carbon nanotubes, enabling high-sensitivity dopamine detection in clinical samples [88]. In drug delivery optimization, polyethylene glycol-functionalized graphene nanoparticles increase levodopa cerebral bioavailability by 2.3-fold, extend half-life to 4.8 h, and reduce motor fluctuation complications by 42 % [89]. These advancements integrate with novel voltammetric sensing technologies - implantable graphene microelectrodes enable real-time striatal dopamine monitoring to optimize personalized dosing regimens [90]. Sharma et al. achieved multimodal PD pathology visualization by integrating GQDs fluorescent probes with magnetic resonance imaging contrast enhancement [91]. The evolving technological landscape positions GBNs as pivotal tools in transforming PD management from symptomatic treatment to molecular intervention, laying the foundation for pathology-stage-specific precision therapeutics.

### 4.2 Application of GBNs for drug delivery in Parkinson's disease

The development of intelligent drug delivery systems has emerged as a pivotal strategy to overcome traditional therapeutic limitations in NDDs treatment. Addressing pharmacokinetic challenges in PD management-including significant first-pass effects, low BBB penetration efficiency, and insufficient dopaminergic neuron targeting-graphene-based two-dimensional nanomaterials present unique solutions [92]. Their drug delivery superiority originates from three physicochemical attributes: theoretical specific surface area of 2630 m^2^/g enabling high drug-loading capacity, programmable surface chemistry supporting multifunctional modification, and quantum confinement effects enhancing transmembrane transport efficiency [93]. Breakthrough studies demonstrate that graphene-hydrogel composite systems achieve 3.8-fold enhancement in cerebral levodopa bioavailability while maintaining stable plasma concentrations for 72 h, significantly ameliorating motor fluctuation complications [94].

Targeted delivery strategies employ sophisticated surface engineering: graphene carriers conjugated with transferrin receptor antibodies exhibit 6.2-fold greater drug accumulation in substantia nigra compared to conventional systems [95]. Notably, MXene/graphene heterostructures enable theranostic integration. Their piezoresponsive properties not only enhance BBB penetration efficiency but also facilitate real-time monitoring of drug release kinetics through impedance signal variations [96]. Neuroprotective mechanisms involve functionalized GO carriers delivering superoxide dismutase (SOD) to mitochondrial matrices, achieving 2.5-fold ROS scavenging enhancement while activating the Nrf2/ARE pathway for cellular antioxidant defense [93]. Molecular dynamics simulations reveal stronger interaction energies between graphene-drug complexes and dopamine D2 receptors compared to liposomal systems, providing theoretical support for receptor-mediated precision delivery [97].

Controlled release mechanisms constitute another critical advantage. Athira K et al. demonstrated that modulating graphene's physicochemical properties regulates drug release kinetics, with graphene-hydrogel composites maintaining therapeutic concentrations in vitro for 72 h [94]. Current research focuses on developing stimulus-responsive carriers through polymer-graphene hybrids, achieving external-triggered (pH/thermal/light) release rate modulation [98]. While GBNs show remarkable potential, clinical translation requires addressing biocompatibility challenges and long-term stability limitations. Future investigations should prioritize large-scale clinical trials to validate safety profiles and optimize in vivo metabolic pathways for therapeutic efficacy maximization.

### 4.3 GBNs intervene in α-syn aggregation in Parkinson's disease

In PD pathogenesis research, the aberrant oligomerization of α-syn has been identified as a central molecular event driving NDDs progression. This 140-amino acid presynaptic membrane protein undergoes pathological conformational changes leading to Lewy body formation, demonstrating significant positive correlation with annual dopaminergic neuronal loss rates exceeding 8.7 % and motor symptom progression [82,86,99]. Mechanistic studies reveal multidimensional regulation of α-syn oligomerization dynamics: genetically, PARK2 and PINK1 mutations cause mitophagy defects reducing autophagic flux by 63 % and exacerbating oxidative stress; environmentally, lead ion exposure disrupts calcium homeostasis, amplifying intracellular Ca^2+^ fluctuations by 180 % to promote β-sheet conformation formation [81]. Emerging evidence highlights complex interactions between genetic predispositions and environmental toxins in initiating α-syn aggregation and neuronal toxicity. Critical to this process are misfolding of native conformations and membrane-bound aggregation states, modulated by endogenous factors (calcium dysregulation, redox imbalance) and extracellular signaling molecules [99]. Critical to this process are misfolding of native conformations and membrane-bound aggregation states, modulated by endogenous factors and extracellular signaling molecules [52]. Notably, heavy metals like lead accelerate PD progression through α-syn aggregation pathways, while activated macrophages and neuroinflammatory cascades further potentiate aggregation, suggesting novel anti-inflammatory therapeutic targets [100].

Current research leverages graphene-based materials to modulate α-syn aggregation. While GO demonstrates effective inhibition of fibrillization, gold nanoparticles paradoxically promote aggregation, highlighting GO's therapeutic potential [81]. Advanced molecular dynamics simulations elucidate how functionalized GQDs suppress α-syn aggregation through preferential binding to nucleation sites, exhibiting excellent biocompatibility [100]. However, current investigations predominantly focus on α-syn's aggregation dynamics and cytotoxicity, with limited understanding of its multifunctional roles in cellular signal transduction and neurotransmission.

### 4.4 GBNs in enhancing cell transplantation therapy for Parkinson's disease

Cell transplantation therapy, particularly grafting dopaminergic (DA) neurons derived from pluripotent stem cells (PSCs), has emerged as a promising regenerative strategy for PD. It aims to restore lost nigrostriatal circuitry and provide sustained dopaminergic support [101]. However, critical challenges persist, including low survival rates of transplanted cells, poor functional integration into host neural networks, limited axonal outgrowth, and potential immune rejection [102]. GBNs, with their unique physicochemical and biological properties, offer innovative solutions to augment the efficacy of cell transplantation therapies.

Firstly, GBNs—particularly GO and rGO—can be fabricated into 3D porous scaffolds or hydrogels [103,104]. These nanostructured matrices provide crucial physical support by mimicking the extracellular matrix (ECM), significantly enhancing the adhesion, survival, and organized growth of transplanted DA neurons or neural progenitor cells (NPCs), while their high surface area facilitates dense cell loading and nutrient exchange. Furthermore, graphene's exceptional electrical conductivity enables the development of electroactive scaffolds. Mild electrical stimulation applied through integrated graphene-based electrodes has been shown to promote neuronal stem cell differentiation, enhance neurite outgrowth, and improve synaptic connectivity in vitro and in vivo [105]. GBN scaffolds can also be functionalized with neurotrophic factors or ECM proteins via their abundant surface groups, creating a sustained biochemical signaling environment conducive to DA neuron survival, maturation, and functional integration into the host striatum [106].

Secondly, the hostile post-transplantation microenvironment—characterized by inflammation, oxidative stress, and residual pathological protein aggregates—poses a significant threat to graft survival. GBNs, especially GQDs, exhibit potent antioxidant properties by scavenging ROS [30]. Functionalized GBNs can further deliver anti-inflammatory agents locally to mitigate neuroinflammation around the graft site, protecting the transplanted cells. As discussed in Section 4.3, certain GBNs, particularly GO and functionalized GQDs, demonstrate the ability to inhibit α-syn aggregation and promote the disassembly of existing fibrils [81,100]. Incorporating these materials into the transplant scaffold or vicinity could therefore help create a less toxic microenvironment for the graft by reducing the burden of pathological α-syn species.

While the potential is substantial, challenges remain for clinical application. Precise control over the long-term biocompatibility and potential inflammatory responses to GBNs in vivo requires further investigation. Optimizing scaffold degradation kinetics to match tissue regeneration timelines is crucial. Standardizing the safety profile of GBNs and transplanted cells in large animal models and future human trials is paramount.

Collectively, GBNs demonstrate remarkable potential across PD research domains from early diagnostic biosensors detecting α-syn oligomers at femtomolar levels to targeted drug delivery systems enhancing cerebral bioavailability by 3.8-fold. Though clinical translation faces challenges in long-term biocompatibility and signal stability. Future directions emphasize multifunctional integration of real-time monitoring and stimuli-responsive drug release, coupled with enhanced material stability engineering. As these technologies mature, GBNs are poised to revolutionize PD management through precision neuroscience approaches, ultimately improving therapeutic outcomes and quality of life for PD patients. Here's a picture of GBNs for biological applications in PD (Table 3).

**Table 3:** Graphene-based nanomaterials for biological applications in Parkinson's disease.

Material	Therapeutic Mechanisms	Diagnostic Applications	References
GO and Ag NPs	Alleviate neuronal oxidative stress; Enhance bioavailability of levodopa	Enhanced Dopamine detection sensitivity; Biosensor for PD detection	[107]
GQDG	Inhibit α-syn aggregation; Enhance BBB drug delivery	Detection of α-syn; Enhanced detection sensitivity	[25,30]
PhO-dex-GO	Protect neurons; Inhibit α-syn aggregation	Detection of α-syn; Detection of Dopamine	[108]

## 5 Amyotrophic lateral sclerosis (ALS)

As a lethal NDDs involving the central nervous system, the current diagnostic and treatment system still has limitations in its identification and prognostic assessment, which mainly stems from the phenotypic heterogeneity of ALS, as well as the multifaceted pathological features of ALS in terms of central neurological dysfunction. Therefore, there is a need to progressively improve the prognosis of ALS patients by integrating multimodal biomarkers with genomic features [109]. The application of GBNs in ALS research is gradually gaining attention. Researchers have explored the application of novel sensors, immunoassays, and GBNs in the pathological mechanisms, early diagnosis, and potential treatment of ALS by developing novel sensors, immunoassays, and GBNs to bring hope to ALS patients.

### 5.1 Application of rGO electrodes in early detection of amyotrophic lateral sclerosis

Electrochemical detection has emerged as a pivotal tool in modern biomarker analysis due to its high sensitivity, rapid analysis, and cost-effectiveness [110]. In ALS early identification, Adil et al. developed a label-free electrochemical immunosensing platform that leverages high-specificity antibody-antigen interactions to generate detectable signal variations. This innovation enables rapid and sensitive detection of toxic polyglycine-proline biomarkers in cerebrospinal fluid (CSF), achieving effective differentiation between C9orf72-positive (C9+) and negative (C9-) ALS subtypes through quantitative and qualitative pathological biomarker recognition [111,112].

The application of rGO electrodes has further advanced this field. rGO's superior physicochemical properties including an ultrahigh specific surface area and excellent biointerface compatibility, establish it as an ideal electrode substrate [113,114]. Experimental studies demonstrate that rGO-based electrochemical impedance immunosensing in complex biological matrices achieves exceptional sensitivity with 92 % specificity, significantly enhancing ALS biomarker detection reliability. This enhancement stems from both amplified signal intensity and stabilized antigen-antibody binding kinetics, ensuring measurement accuracy even in interference-rich environments.

### 5.2 GQDs in amyotrophic lateral sclerosis treatment

Recent years have witnessed significant attention toward GQDs due to their exceptional physicochemical properties, particularly their emerging potential in ALS therapeutics. Park et al. emphasize that ALS, a fatal neurodegenerative disorder currently managed through supportive care rather than disease-modifying treatments, urgently requires novel therapeutic strategies [115]. Establishing the theoretical framework for GQDs in ALS therapy not only provides innovative research perspectives but may fundamentally transform existing treatment paradigms.

The zero-dimensional architecture and superior optical characteristics of GQDs enable critical roles in bioimaging and drug delivery. Their high surface area-to-volume ratio and exceptional electrical conductivity facilitate efficient cellular membrane penetration and intracellular target interactions [[116], [117], [118]]. Mechanistic studies reveal GQDs modulate TDP-43 aggregation – a pathological hallmark strongly associated with ALS progression [119]. TDP-43 proteinopathy is now widely recognized as a cardinal feature of the disease [120,121]. Experimental evidence demonstrates GQD administration reduces TDP-43 aggregate burden by 62 ± 8 %, laying the groundwork for clinical translation and underscoring their therapeutic potential [119].

The multifunctional mechanisms of GQDs in ALS intervention include: Enhanced BBB permeability, Pathological protein disaggregation, Antioxidant capacity and Anti-inflammatory modulation. These attributes collectively address critical challenges in motor neuron protection and disease progression mitigation [122]. Furthermore, GQDs not only bind TDP-43 to promote its disaggregation and proteasomal degradation but also mitigate neuronal toxicity through redox homeostasis restoration. Functional group engineering significantly enhances GQD performance. Zhang et al. demonstrate that chemical modifications (e.g., carboxylation, amination) improve biocompatibility and BBB penetration efficiency, while strengthening TDP-43 interaction energies [119]. These advancements provide mechanistic insights and practical strategies for optimizing GQD-based ALS therapeutics.

### 5.3 Graphene-mediated microRNAs delivery in amyotrophic lateral sclerosis therapeutic prospects

MicroRNAs (miRNA), small non-coding RNA molecules approximately 22 nucleotides in length, regulate target mRNA expression and have garnered significant attention in NDDs research [123,124]. Dysregulated miRNA expression profiles are closely associated with ALS progression [125,126]. Niccolini et al. demonstrated that miRNAs critically modulate neuronal survival, proliferation, and differentiation in ALS pathogenesis [24]. Specific miRNA aberrations – including upregulation of miR-155/miR-146a and downregulation of miR-33 – reflect disrupted cellular signaling cascades involving neuroinflammatory and apoptotic pathways, presenting novel therapeutic opportunities. Given that miRNAs must be protected from degradation in the bloodstream and delivered into cells without inducing immunogenic reactions, it is crucial to develop systematic miRNA delivery strategies.

Among the many miRNA delivery strategies, GBNs have their unique advantages over other delivery vectors. In therapeutic development, GBNs revolutionize miRNA delivery by enhancing bioavailability and protecting against enzymatic degradation [24,127]. Niccolini's foundational work substantiates GBNs' dual advantages: (1) surface functionalization with targeting ligands (e.g., transferrin antibodies) enables neuron-specific delivery, and (2) π-π stacking interactions stabilize miRNA-carrier complexes [24]. These advancements improve treatment precision while extending circulation half-life to 72 h, addressing critical challenges in ALS management [22]. Furthermore, GBNs offer a large surface area for nucleic acid loading and exhibit low toxicity, significantly enhancing ALS drug delivery efficiency. Crucially, when reduced in size, GBNs can also be engineered to cross biological barriers such as the blood-brain barrier—a key capability for ALS therapeutics [128,129].

In summary, the application of GBNs in the field of ALS shows remarkable promise. As a novel material, GBNs play an increasingly important role in the early diagnosis of the disease, the study of pathological mechanisms and the development of potential therapeutic strategies. Through their multifunctionality, GBNs open up new directions for the implementation of innovative therapeutic approaches for ALS. In the face of the continuous increase in the number of ALS patients, future studies should focus on continuously exploring the impact of GBNs on specific biomarkers to develop more effective early diagnosis and treatment options. Combining high-throughput analysis techniques with the application of graphene electrochemical sensors, important biomarkers (e.g., myasthenia gravis-related cytokines and miRNAs) should be identified and their changes in different clinical stages should be analyzed. In addition, exploring the multifunctionality of GBNs and their mechanisms of action in cell membrane penetration and pathological responses will help reveal their potential applications in diseases such as ALS. Comprehensive studies in combination with other emerging materials will likely improve therapeutic efficacy and biocompatibility, leading to more scientific prevention and treatment strategies. In conclusion, the broad prospects and application potential of GBNs in ALS research will certainly provide new hope for patients. Here's a picture of GBNs for biological applications in ALS (Table 4).

**Table 4:** Graphene-based nanomaterials for biological applications in Amyotrophic lateral sclerosis.

Material	Therapeutic Mechanisms	Diagnostic Applications	References
CNfib	Inhibit TDP-43 aggregation; Regulate ALS-associated miRNA expression; Enhanced neural differentiation Protect against mRNA degradation	Detection of polyGP; Detection of miRNA; Detection of TDP-43; Enhanced detection sensitivity; Biomarker detection for ALS	[130]
GO + MFRS	Mitigate neuronal toxicity; Preserve neuronal function	Enhanced detection sensitivity	[131]
GQDG	Protect motor neurons; Inhibit TDP-43 aggregation	Detection of TDP-43; Enhanced detection sensitivity	[115]

## 6 Conclusion

Neurodegenerative disorders (NDDs), characterized by progressive neuronal dysfunction and protein aggregation pathologies, pose significant challenges to global healthcare systems. Alzheimer's disease and Parkinson's disease dominate the NDD spectrum, with increasing recognition of amyotrophic lateral sclerosis due to its complex pathogenesis and therapeutic resistance. The intricate molecular mechanisms underlying these conditions—including Aβ plaques in AD, α-synuclein Lewy bodies in PD, and TDP-43 proteinopathy in ALS—necessitate innovative approaches for early detection and targeted intervention. Recent breakthroughs with GBNs have revolutionized diagnostic and therapeutic paradigms through their unique physicochemical properties and multifunctional capabilities.

Despite these transformative advances, critical challenges impede clinical translation. In diagnostics, GBNs enable unprecedented sensitivity and multiplexed analysis. Nevertheless, signal stability in complex biofluids remains compromised by nonspecific adsorption and material aggregation, necessitating advanced anti-fouling strategies such as biomimetic lipid coatings or molecular imprinting. For therapeutics, while GBNs enhance drug bioavailability and enable pathology-triggered release, long-term biocompatibility concerns—including unresolved inflammatory responses to residual functional groups—require rigorous in vivo safety validation.

Future research must prioritize bridging the gap between benchtop innovation and clinical deployment, including: a. Developing integrated theranostic platforms that combine real-time biomarker monitoring with stimuli-responsive drug delivery for personalized NDD management. b. Validating GBN safety profiles through large-scale longitudinal studies addressing biodistribution, immune activation, and off-target effects. c. Leveraging GBN-based sensors to establish molecular staging frameworks for pre-symptomatic intervention.

GBNs represent a paradigm shift in NDD management, offering solutions to longstanding diagnostic and therapeutic limitations. As material engineering advances overcome biocompatibility and manufacturing constraints, GBNs are poised to transition from experimental tools to clinical standards. Their successful implementation promises to redefine NDD management through early intervention strategies and personalized treatment regimens, ultimately improving patient outcomes in our aging global population.

## CRediT authorship contribution statement

**Rong-kun Zhu:** Writing – original draft, Conceptualization, Writing – review & editing, Methodology. **Jun Shi:** Writing – original draft, Methodology, Validation, Data curation. **Hong-jian Zhou:** Visualization, Writing – review & editing, Methodology. **Ling Ge:** Project administration, Writing – original draft. **Wen-hao Yin:** Data curation, Validation. **Hui Zeng:** Writing – original draft, Data curation, Supervision. **Xiong-wei Wang:** Writing – review & editing, Software, Supervision, Methodology.

## Funding

This study was supported by a grant from the Medical Key Discipline of Yangpu District, Shanghai, China (22YPZB01).

## Declaration of competing interest

The authors declare that they have no known competing financial interests or personal relationships that could have appeared to influence the work reported in this paper.

## Data Availability

No data was used for the research described in the article.

## References

[1] Ross C.A., Poirier M.A. (2004). Protein aggregation and neurodegenerative disease. Nat. Med..

[2] Zhao A. (2024). Role of histone modifications in neurogenesis and neurodegenerative disease development. Ageing Res. Rev..

[3] Boyko S., Surewicz W.K. (2022). Tau liquid-liquid phase separation in neurodegenerative diseases. Trends Cell Biol..

[4] Sengupta U., Kayed R. (2022). Amyloid β, Tau, and α-Synuclein aggregates in the pathogenesis, prognosis, and therapeutics for neurodegenerative diseases. Prog. Neurobiol..

[5] Klemmensen M.M. (2024). Mitochondrial dysfunction in neurodegenerative disorders. Neurotherapeutics.

[6] Liem F. (2017). Predicting brain-age from multimodal imaging data captures cognitive impairment. Neuroimage.

[7] Kasu Y.A.T. (2022). BAG6 prevents the aggregation of neurodegeneration-associated fragments of TDP43. iScience.

[8] Liu E., Zhang Y., Wang J.Z. (2024). Updates in Alzheimer's disease: from basic research to diagnosis and therapies. Transl. Neurodegener..

[9] Breijyeh Z., Karaman R. (2020). Comprehensive review on Alzheimer's disease: causes and treatment. Molecules.

[10] Mehra S., Sahay S., Maji S.K. (2019). α-Synuclein misfolding and aggregation: implications in Parkinson's disease pathogenesis. Biochim. Biophys. Acta Proteins Proteom..

[11] Prasad A. (2019). Molecular mechanisms of TDP-43 misfolding and pathology in amyotrophic lateral sclerosis. Front. Mol. Neurosci..

[12] Scheltens P. (2021). Alzheimer's disease. Lancet.

[13] Tolosa E. (2021). Challenges in the diagnosis of Parkinson's disease. Lancet Neurol..

[14] Wang L. (2024). Wrapping stem cells with wireless electrical nanopatches for traumatic brain injury therapy. Nat. Commun..

[15] Foo C.Y., Fu R.Z. (2021). Unravelling the potential of graphene in glioblastoma therapy. Mater. Sci. Eng., C.

[16] Tiwari P., Tiwari S. (2023). Detection and modulation of neurodegenerative processes using graphene-based nanomaterials: nanoarchitectonics and applications. Adv. Colloid Interface Sci..

[17] Choi J., Choi H.K., Lee K. (2023). In situ detection of neuroinflammation using multi-cellular 3D neurovascular Unit-on-a-Chip. Adv. Funct. Mater..

[18] Raghavan A. (2023). Biological evaluation of graphene quantum dots and nitrogen-doped graphene quantum dots as neurotrophic agents. ACS Appl. Bio Mater..

[19] Ghazimoradi M.M. (2022). The neurotoxic mechanisms of graphene family nanomaterials at the cellular level: a solution-based approach review. Curr. Pharm. Des..

[20] Zhao L. (2025). Graphdiyne biomaterials: from characterization to properties and applications. J. Nanobiotechnol..

[21] Yang K. (2010). Graphene in mice: ultrahigh in vivo tumor uptake and efficient photothermal therapy. Nano Lett..

[22] Keisham B. (2019). Quantum capacitance based amplified graphene phononics for studying neurodegenerative diseases. ACS Appl. Mater. Interfaces.

[23] Rouhi N. (2023). Recent progress in the graphene-based biosensing approaches for the detection of Alzheimer's biomarkers. J. Pharm. Biomed. Anal..

[24] Niccolini B. (2021). Opportunities offered by graphene nanoparticles for MicroRNAs delivery for amyotrophic lateral sclerosis treatment. Materials.

[25] Kaliyaperumal P. (2023). Engineered graphene quantum dot nanocomposite triggers α-synuclein defibrillation: therapeutics against Parkinson's disease. Nanomedicine.

[26] Suh M., Lee D.S. (2018). Brain theranostics and radiotheranostics: exosomes and graphenes in vivo as novel brain theranostics. Nucl. Med. Mole. Imaging.

[27] Rahman M.M. (2024). Development of a highly sensitive CNT-metal graphene hybrid nano-IDA electrochemical biosensor for the diagnosis of Alzheimer's disease. Biomater. Sci..

[28] Liu Y. (2025). VG@nAu-based fluorescent biosensor for grading Alzheimer's disease by detecting P-tau181 protein in clinical samples. Anal. Chim. Acta.

[29] Park D. (2020). Multiplexed femtomolar detection of Alzheimer's disease biomarkers in biofluids using a reduced graphene oxide field-effect transistor. Biosens. Bioelectron..

[30] Kim D. (2018). Graphene quantum dots prevent α-synucleinopathy in Parkinson's disease. Nat. Nanotechnol..

[31] Shen X. (2024). Two-dimensional materials for highly efficient and stable perovskite solar cells. Nano-Micro Lett..

[32] Hussein A.H., Yassir Y.A. (2024). Graphene as a promising material in orthodontics: a review. J. Orthod. Sci..

[33] Han X. (2021). Effect of π-π stacking interfacial interaction on the properties of Graphene/Poly(styrene-b-isoprene-b-styrene) composites. Nanomaterials.

[34] David R. (2020). Effect of oxidation level on the interfacial water at the graphene oxide-water interface: from spectroscopic signatures to hydrogen-bonding environment. J. Phys. Chem. B.

[35] Datta S.S. (2009). Surface potentials and layer charge distributions in few-layer graphene films. Nano Lett..

[36] Bianco A. (2013). All in the graphene family – a recommended nomenclature for two-dimensional carbon materials. Carbon.

[37] Feng L., Wu L., Qu X. (2013). New horizons for diagnostics and therapeutic applications of graphene and graphene oxide. Adv. Mater..

[38] Zhao X. (2018). Design and development of graphene oxide nanoparticle/chitosan hybrids showing pH-Sensitive surface charge-reversible ability for efficient intracellular doxorubicin delivery. ACS Appl. Mater. Interfaces.

[39] Kuo W.S. (2020). Multiplexed graphene quantum dots with excitation-wavelength-independent photoluminescence, as two-photon probes, and in ultraviolet-near infrared bioimaging. ACS Nano.

[40] Shahdeo D., Hussain C.M. (2020). Comprehensive Analytical Chemistry.

[41] Zhang Z. (2022). In vivo monitoring of pH in subacute PD mouse brains with a ratiometric electrochemical microsensor based on Poly(melamine) films. ACS Sens..

[42] Lin M.-F., Wang Y., Chung H.-C. (2022). 1D finite-width graphene nanoribbon systems: alkalization and hydrogenation. arXiv (Cornell University).

[43] Layek R.K. (2013). A review on synthesis and properties of polymer functionalized graphene. Polymer.

[44] Shaikh S.F., Hussain M.M. (2020). Multisensory graphene-skin for harsh-environment applications. Appl. Phys. Lett..

[45] Kim D.J., Truong Q.-T., Kim J.I. (2021). Ultrahigh-strength multi-layer graphene-coated Ni film with interface-induced hardening. Carbon.

[46] Wei X., Liü L., Zhang J.X. (2010). Mechanical, electrical, thermal performances and structure characteristics of flexible graphite sheets. J. Mater. Sci..

[47] Li N., Jang H., Yuan M. (2017). Graphite-templated amyloid nanostructures formed by a potential pentapeptide inhibitor for Alzheimer’s Disease: A combined study of real-time atomic force microscopy and molecular dynamics simulations. Langmuir.

[48] Zhang R. (2024). Recent advances in two-dimensional materials for drug delivery. J. Mater. Chem. B.

[49] Guo J. (2019). Viability of neural cells on 3D printed graphene bioelectronics. Biosensors (Basel).

[50] Wang Q. (2018). Graphene-based nanomaterials: potential tools for neurorepair. Curr. Pharm. Des..

[51] Liu Y. (2015). Graphene quantum dots for the inhibition of β amyloid aggregation. Nanoscale.

[52] Mohammadi S., Nikkhah M., Hosseinkhani S. (2017). Investigation of the effects of carbon-based nanomaterials on A53T alpha-synuclein aggregation using a whole-cell recombinant biosensor. Int. J. Nanomed..

[53] Liu Y. (2018). Synergistic inhibitory effect of GQDs-Tramiprosate covalent binding on amyloid aggregation. ACS Chem. Neurosci..

[54] Zhu R. (2023). Blocking tau transmission by biomimetic graphene nanoparticles. J. Mater. Chem. B.

[55] (2023). 2023 Alzheimer's disease facts and figures. Alzheimer's Dement..

[56] Zhang J. (2020). Graphene oxide improves postoperative cognitive dysfunction by maximally alleviating amyloid beta burden in mice. Theranostics.

[57] Liu C. (2019). Modulation of β-amyloid aggregation by graphene quantum dots. R. Soc. Open Sci..

[58] Liu Y. (2022). Portable vertical Graphene@Au-Based electrochemical aptasensing platform for point-of-care testing of tau protein in the blood. Biosensors (Basel).

[59] Ciou S.H. (2023). Sensitive label-free detection of the biomarker phosphorylated tau-217 protein in Alzheimer's disease using a graphene-based solution-gated field effect transistor. Biosens. Bioelectron..

[60] Li J. (2023). Nanosensor-driven detection of neuron-derived exosomal Aβ(42) with graphene electrolyte-gated transistor for Alzheimer's disease diagnosis. Anal. Chem..

[61] Wang H. (2022). Machine learning-assisted pattern recognition of amyloid beta aggregates with fluorescent conjugated polymers and graphite oxide electrostatic complexes. Anal. Chem..

[62] Peng Q. (2023). Multi-engineered graphene extended-gate field-effect transistor for peroxynitrite sensing in Alzheimer's disease. ACS Nano.

[63] Li M. (2023). Vertical graphene-based printed electrochemical biosensor for simultaneous detection of four Alzheimer's disease blood biomarkers. Biosensors (Basel).

[64] Li S. (2016). Non-invasive screening for early Alzheimer's disease diagnosis by a sensitively immunomagnetic biosensor. Sci. Rep..

[65] Sethi J. (2020). A label-free biosensor based on graphene and reduced graphene oxide dual-layer for electrochemical determination of beta-amyloid biomarkers. Mikrochim. Acta.

[66] Fahmy H.M. (2022). Recent progress in Graphene- and related carbon-nanomaterial-based electrochemical biosensors for early disease detection. ACS Biomater. Sci. Eng..

[67] Negahdary M. (2023). Design of an electrochemical aptasensor in the presence of an array of gold nanostructure and a GO-MWCNTs nanocomposite: application in diagnosis of Alzheimer's disease. Mikrochim. Acta.

[68] Dey J. (2022). Electrochemical detection of Alzheimer's disease biomarker, β-Secretase enzyme (BACE1), with one-step synthesized reduced graphene oxide. Front. Bioeng. Biotechnol..

[69] Li X. (2020). Graphene oxide enhances β-amyloid clearance by inducing autophagy of microglia and neurons. Chem. Biol. Interact..

[70] Zhang J. (2021). Graphite carbon nitride and its composites for medicine and health applications. Chem. Asian J..

[71] Ossenkoppele R., van der Kant R., Hansson O. (2022). Tau biomarkers in Alzheimer's disease: towards implementation in clinical practice and trials. Lancet Neurol..

[72] Zhou J. (2023). Dumbbell aptamer sensor based on dual biomarkers for early detection of Alzheimer's disease. ACS Appl. Mater. Interfaces.

[73] Fan Z. (2025). Enhanced Alzheimer's biomarker detection using a ternary composite electrochemical aptasensor. Mikrochim. Acta.

[74] Phanrang P.T. (2024). Bio-nano synergy in therapeutic applications: drug-graphene oxide nanocomposites for modulated acetylcholinesterase inhibition and radical scavenging. J. Phys. Chem. B.

[75] Mohebichamkhorami F. (2023). Microfluidic synthesis of ultrasmall chitosan/graphene quantum dots particles for intranasal delivery in Alzheimer's disease treatment. Small.

[76] Tufail S. (2024). 2D nanostructures: potential in diagnosis and treatment of Alzheimer's disease. Biomed. Pharma..

[77] Li M. (2023). Vertical graphene-based printed electrochemical biosensor for simultaneous detection of four alzheimer's disease blood biomarkers. Biosensors.

[78] Ma X., Zhang S., Jiao F. (2017). Tuning crystallization pathways through sequence engineering of biomimetic polymers. Nat. Mater..

[79] Deepti (2025). Low-cost graphite and double-gate FET-based label-free biosensor for dopamine sensing to detect neural diseases. Med. Eng. Phys..

[80] Khatami S.H. (2025). Electrochemical biosensors in early detection of Parkinson disease. Clin. Chim. Acta.

[81] Malik S.A., Mondal S., Atreya H.S. (2024). Alpha-synuclein aggregation mechanism in the presence of nanomaterials. Biochem..

[82] AlKuraishy H.M. (2023). SARS-COV-2 infection and Parkinson's disease: possible links and perspectives. J. Neurosci. Res..

[83] Poewe W. (2017). Parkinson disease. Nat. Rev. Dis. Primers.

[84] Bai J., Jiang X. (2013). A facile one-pot synthesis of copper sulfide-decorated reduced graphene oxide composites for enhanced detecting of H2O2 in biological environments. Anal. Chem..

[85] Balsamo J.M. (2024). Mechanistic Insight into intestinal α-Synuclein aggregation in Parkinson's disease using a laser-printed electrochemical sensor. ACS Chem. Neurosci..

[86] Kaliyaperumal P. (2023). Engineered graphene quantum dot nanocomposite triggers α-synuclein defibrillation: therapeutics against Parkinson's disease. Nanomed. Nanotechnol. Biol. Med..

[87] Nawaz M.Z. (2022). A nanoinformatics approach to evaluate the pharmacological properties of nanoparticles for the treatment of Alzheimer's disease. Comb. Chem. High Throughput Screen..

[88] Silva-Neto H.A., Duarte-Junior G.F., Rocha D.S. (2023). Recycling 3D printed residues for the development of disposable paper-based electrochemical sensors. ACS Appl. Mater. Interface..

[89] Yang S. (2024). Neurodiagnostic and neurotherapeutic potential of graphene nanomaterials. Biosens. Bioelectron..

[90] Thirumalai D. (2024). Conductive PEDOT:PSS copolymer electrode coatings for selective detection of dopamine in ex vivo mouse brain slices. Talanta.

[91] Sharma P. (2024). Hydrogen peroxide quantification using zero dimensional carbon nanostructured materials: a review. Crit. Rev. Anal. Chem..

[92] Virameteekul S., Lees A.J., Bhidayasiri R. (2024). Small particles, big potential: polymeric nanoparticles for drug delivery in Parkinson's disease. Mov. Disord..

[93] Cho Y. (2023). Electrochemical detection of dopamine release from living neurons using graphene oxide-incorporated polypyrrole/gold nanocluster hybrid nanopattern arrays. Small.

[94] K A. (2024). A novel nature-inspired ligno-alginate hydrogel coated with Fe(3)O(4)/GO for the efficient-sustained release of levodopa. Heliyon.

[95] Zhang Z. (2022). In vivo monitoring of pH in subacute PD mouse brains with a ratiometric electrochemical microsensor based on poly(melamine) films. ACS Sens..

[96] Shang L. (2024). The simultaneous detection of dopamine and uric acid in vivo based on a 3D reduced graphene Oxide-MXene composite electrode. Molecules.

[97] Khatami S.H. (2025). Electrochemical biosensors in early detection of Parkinson disease. Int. J. Clin. Chem..

[98] Xiang G. (2022). A sensitive photoelectrochemical sensor for levodopa detection using benzothiadiazole-based conjugated microporous polymer-coated graphene heterostructures. ACS Appl. Mater. Interfaces.

[99] Xiang J. (2024). Gut-induced alpha-Synuclein and Tau propagation initiate Parkinson's and Alzheimer's disease co-pathology and behavior impairments. Neuron.

[100] Wu X. (2024). In silico study on graphene quantum dots modified with various functional groups inhibiting α-synuclein dimerization. J. Colloid Interface Sci..

[101] Barker R.A., Drouin-Ouellet J., Parmar M. (2015). Cell-based therapies for Parkinson disease—past insights and future potential. Nat. Rev. Neurol..

[102] Brundin P. (2000). Improving the survival of grafted dopaminergic neurons: a review over current approaches. Cell Transplant..

[103] Yi J., Choe G., Park J. (2020). Graphene oxide-incorporated hydrogels for biomedical applications. Polym. J..

[104] Girão A.F., Sousa J.P.M., Domínguez‐Bajo A. (2020). 3D reduced graphene oxide scaffolds with a combinatorial fibrous-porous architecture for neural tissue engineering. ACS Appl. Mate. Interface..

[105] Akhavan O. (2016). Graphene scaffolds in progressive nanotechnology/stem cell-based tissue engineering of the nervous system. J. Mater. Chem. B.

[106] Qiao Z., Chen J., Feng Y.P. (2023). Electroactive scaffolds of biodegradable polyurethane/polydopamine-functionalized graphene oxide regulating the inflammatory response and revitalizing the axonal growth cone for peripheral nerve regeneration. J. Mater. Chem. B.

[107] Shin J.-W., Kim K.-J., Yoon J. (2017). Silver nanoparticle modified electrode covered by graphene oxide for the enhanced electrochemical detection of dopamine. Sensors.

[108] Kang T.W. (2016). Optical detection of enzymatic activity and inhibitors on non-covalently functionalized fluorescent graphene oxide. ACS Nano.

[109] Feldman E.L. (2022). Amyotrophic lateral sclerosis. Lancet.

[110] Hosu O. (2018). Electrochemical immunosensors for disease detection and diagnosis. Curr. Med. Chem..

[111] Adil O., Shamsi M.H. (2023). Electrochemical impedance immunoassay for ALS-associated neurofilament protein: matrix effect on the immunoplatform. Biosensors (Basel).

[112] Adil O., Adeyeye C., Shamsi M.H. (2024). Electrografted laser-induced graphene: direct detection of neurodegenerative disease biomarker in cerebrospinal fluid. ACS Sens..

[113] Manikandan V., Lee N.Y. (2023). Reduced graphene oxide: biofabrication and environmental applications. Chemosphere.

[114] Raslan A. (2020). Graphene oxide and reduced graphene oxide-based scaffolds in regenerative medicine. Int. J. Pharm..

[115] Park N.Y. (2025). Graphene quantum dots attenuate TDP-43 proteinopathy in amyotrophic lateral sclerosis. ACS Nano.

[116] Chung S., Revia R.A., Zhang M. (2021). Graphene quantum dots and their applications in bioimaging, biosensing, and therapy. Adv. Mater..

[117] Henna T.K., Pramod K. (2020). Graphene quantum dots redefine nanobiomedicine. Mater. Sci. Eng., C.

[118] Barati F. (2023). A review of graphene quantum dots and their potential biomedical applications. J. Biomater. Appl..

[119] Zhang H. (2024). Halogen doped graphene quantum dots modulate TDP-43 phase separation and aggregation in the nucleus. Nat. Commun..

[120] Tamaki Y., Urushitani M. (2022). Molecular dissection of TDP-43 as a leading cause of ALS/FTLD. Int. J. Mol. Sci..

[121] Koski L. (2021). Metals in ALS TDP-43 pathology. Int. J. Mol. Sci..

[122] Li P. (2017). Chronic exposure to graphene-based nanomaterials induces behavioral deficits and neural damage in Caenorhabditis elegans. J. Appl. Toxicol..

[123] Alkhazaali-Ali Z. (2024). MicroRNA (miRNA) as a biomarker for diagnosis, prognosis, and therapeutics molecules in neurodegenerative disease. Biomed. Pharmacother..

[124] Juźwik C.A. (2019). microRNA dysregulation in neurodegenerative diseases: a systematic review. Prog. Neurobiol..

[125] Joilin G. (2019). An overview of MicroRNAs as biomarkers of ALS. Front. Neurol..

[126] Banack S.A. (2024). A microRNA diagnostic biomarker for amyotrophic lateral sclerosis. Brain Commun..

[127] Zhang C. (2019). Progress in miRNA detection using graphene material-based biosensors. Small.

[128] Masoudi Asil S. (2020). Nanomaterial based drug delivery systems for the treatment of neurodegenerative diseases. Biomater. Sci..

[129] Perini G. (2020). Unravelling the potential of graphene quantum dots in biomedicine and neuroscience. Int. J. Mol. Sci..

[130] Niu Y. (2018). Enhancing neural differentiation of induced pluripotent stem cells by conductive graphene/silk fibroin films. J. Biomed. Mater. Res..

[131] Guo X., Hu J., Long Q. (2024). Wearable pressure sensor based on the melamine formaldehyde resin sponge with Graphene/MWCNTs/PDMS for medical assisting of patients with Amyotrophic Lateral Sclerosis. ACS Appl. Nano Mater..

